# The unsolved problem of musculoskeletal hydatid disease: two case reports

**DOI:** 10.1186/s13256-023-04275-4

**Published:** 2023-12-27

**Authors:** Philani Ntombela, Zweli Linda, Tiego Hlapolosa, Maxwell Jingo

**Affiliations:** 1 Department of Orthopaedics, University of the Witwatersrand, 7 York Road Park-Town, Johannesburg, South Africa; 2 Orthopaedic Oncology and Infections Unit, Charlotte Maxeke Johannesburg Academic Hospital, University of the Witwatersrand, 7 York Road, Park-Town, Johannesburg, 2193 South Africa

**Keywords:** Hydatid disease, Hydatidosis, Pelvis, Musculoskeletal

## Abstract

**Background:**

Hydatidosis is a parasitic infection caused by Echinococcus granulosus and humans are usually an accidental intermediate host. Involvement of the musculoskeletal system is reported to occur in 0.5% to 4% of the cases.

**Case presentation:**

We present our experience with two cases of musculoskeletal hydatidosis in black African patients that required orthopaedic surgical intervention. A 51-year-old black African female presented with right hip hydatid disease and a 37-year-old black African female presented with the disease affecting the left shoulder. Both patients presented with joint pain and reduced range of motion. The patient with involvement of the shoulder had a background history of human immunodeficiency virus, this was not present with the other patient. Diagnostic work-up confirmed peri-articular hydatid disease and both patients were surgically managed with arthroplasty. Post-operative complications encountered include hardware loosening from bone lysis and hardware failure.

**Discussion:**

The medical literature describes a limited number of cases of peri-articular musculoskeletal hydatid disease. Patients are often subjected to many investigations, prolonged treatment periods and multiple surgeries. Concurrent use of medical and surgical treatment is advocated however, the choice of surgery is individualised.

**Conclusion:**

Hydatid disease must always feature in the differential diagnosis of multiple lytic bone lesions and radical surgical intervention may be required from the outset.

## Introduction

Hydatidosis is a parasitic infection caused by Echinococcus granulosus and humans are usually an accidental intermediate host [1]. The infestation largely affects the liver and the lung because 70% of the cysts filter into the hepatic sinusoids whilst the rest is through the lungs [1–3]. Involvement of the musculoskeletal system is reported to occur in 0.5% to 4% of the cases [4, 5]. Once at this point, the disease is difficult to treat and cure is even more challenging to attain. There is a link between hydatid disease and the human immunodeficiency virus (HIV) through immunosuppression [6]. Far more common causes of suspicious bone lesions are entertained before a consideration of hydatid disease is made. This can cause significant delays in diagnosis and treatment. There is scarcity of literature on musculoskeletal hydatid disease that lend itself to surgical orthopaedic management [2]. The disease is associated with significant morbidity and high recurrence in orthopaedic patients [2]. Exclusion of the involvement of other organ systems (liver, lung) is crucial and presents an opportunity for radical excision of the disease if it is localized in the musculoskeletal region like the hip [5]. Other than immunosuppression, risk factors for the disease include sheep and cattle arears, exposure to domestic pets, rural dwelling and the female gender [3]. We present our experience with two cases of musculoskeletal hydatidosis that required orthopaedic surgical intervention. Case one is of the disease involving the proximal femur and hemi-pelvis and case 2 is that involving the proximal humerus.

### Case 1

A 51-year-old black African female presented with a painful right hip and inability to ambulate after a low-energy fall. She did not have any chronic medical condition and was HIV negative. She was previously seen 10 years ago with the similar hip pain and reduced range of motion. No contributory psychosocial factors were elicited and the patient was from an urban region in the city.

#### Clinical findings

She mobilised with an antalgic gait and was tender in the right groin. No cutaneous changes were noted and no palpable mass was elicited. She also had reduced hip abduction and internal rotation. Systemic evaluation did not reveal any positive findings. Table 1 demonstrates the timeline of the diagnostic work-up of the patient.

**Table 1:** Timeline of diagnostic work-up and findings

	Radiographs	MRI	Biopsy
2011	Multiple lytic lesions	Cystic lesions	Inconclusive
2021	Multiple lytic lesions	Cystic lesions	Hydatid disease

*MRI* magnetic resonance imaging

#### Diagnostic assessment

A plain radiograph showed multiple lytic lesions in the right proximal femur and hemi-pelvis. Serological tests for hydatid disease were not done as these did not form part of our standard work-up for bone lesions.

A magnetic resonance imaging (MRI) scan was performed followed by a biopsy at that stage which was inconclusive. As part of standard protocol work-up for suspicious bone lesions, a CT scan of the chest and abdomen was performed. This revealed no further lesions. She was suspected of and treated as skeletal tuberculosis (TB) however, after 6 months of treatment she had shown no response. A possible differential of multiple myeloma was excluded by further skeletal survey and negative protein electrophoresis.

#### Therapeutic intervention

Due to a high index of clinical suspicion, she was empirically started on treatment with anti-parasites (albendazole 10 mg/kg/day for 3 months) and bisphosphonates plus protected weight bearing. She was lost to follow-up subsequently before she returned with recurrence of symptoms.

**Fig. 1:**
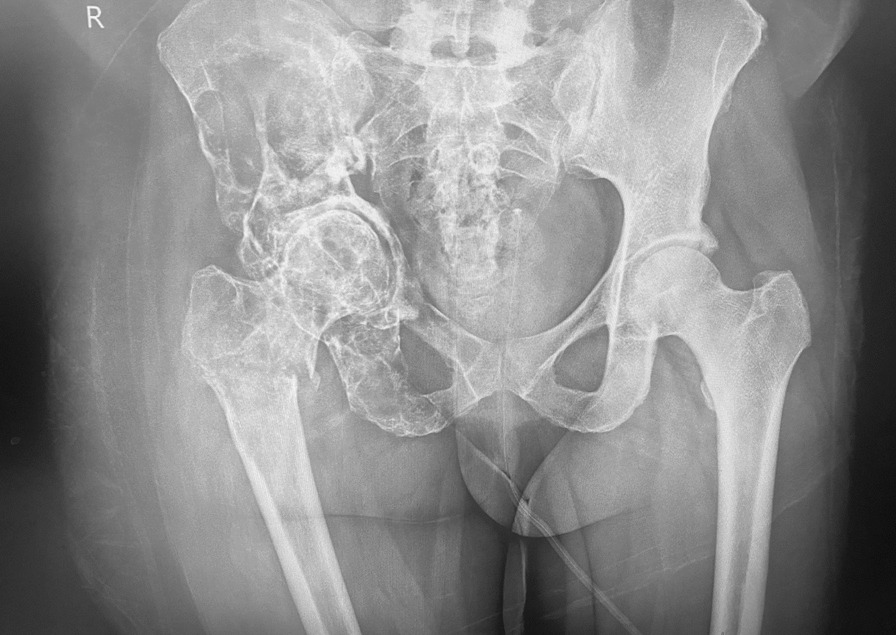
Pre-operative AP view of the pelvis showing multiple lytic/cystic lesions of the right hemi-pelvis + proximal femur. An associated subtrochanteric femur fracture + acetabular protrusion can be seen

**Fig. 2:**
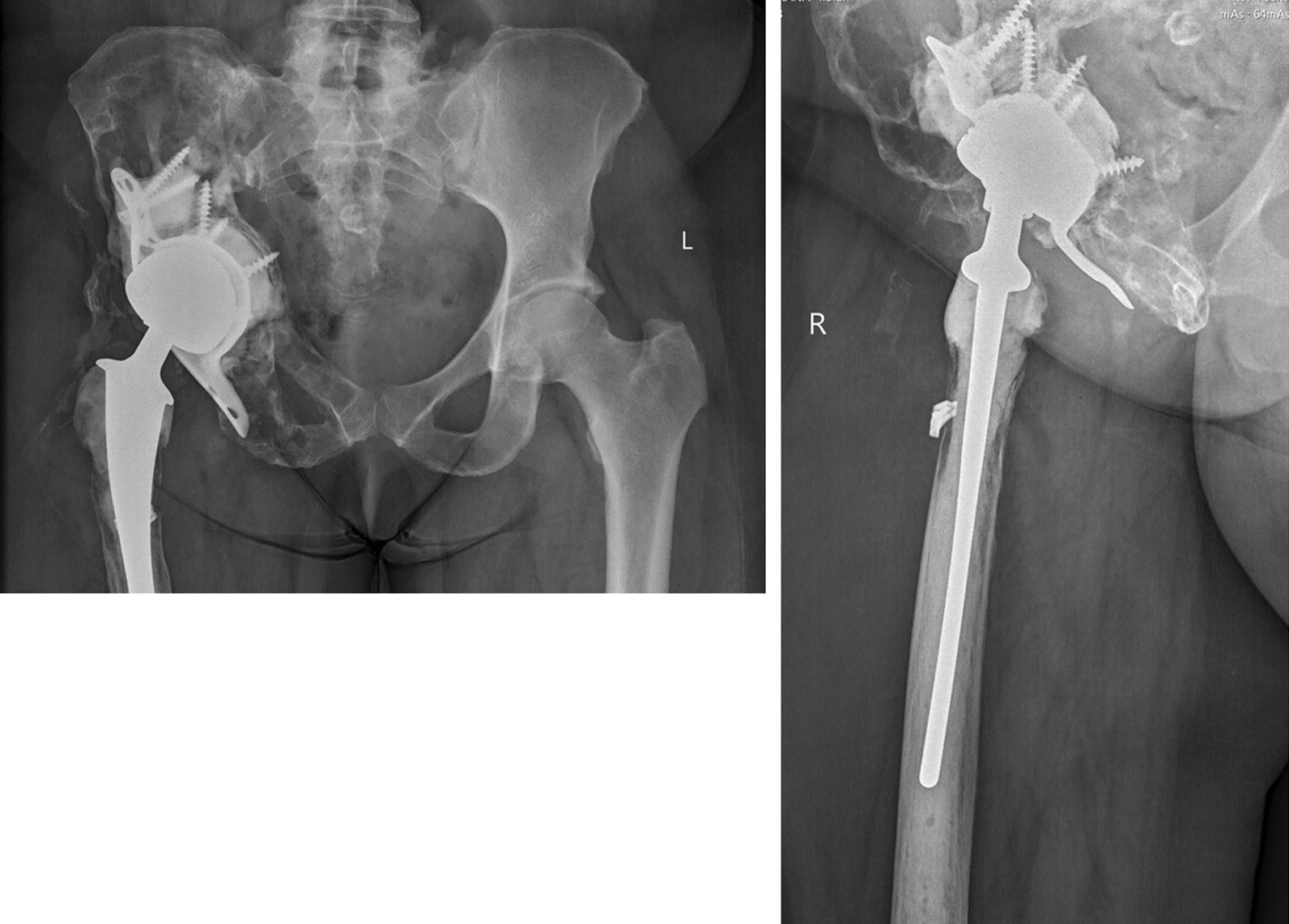
Post-operative AP view of the pelvis and lateral view of the right hip

After this, 10 years (index presentation) later she presented with recurrence of symptoms and a biopsy confirmed hydatid disease by identifying daughter cysts in the section with protoscolices containing a double row of refractile hooklets. Figure 1 shows radiographs at index presentation. She was counselled for a total hip replacement (THR) and was restarted on medical treatment. The surgery was performed and Fig. 2 shows the post-operative radiographs.

#### Outcome

Two years post-operatively she was mobilising with one crutch and the pain was well controlled, however, she now had implant loosening from further lysis of bone and will require revision surgery.

### Case 2

A 37-year-old black African female presented with acute worsening of long-standing left shoulder pain. She reported attempting to lift up a heavy object and the arm gave way. Prior to this, she was on over-the-counter pain medication and had never consulted a medical professional.

She did not have any documented chronic comorbidities but further investigations revealed that she was HIV positive. The systemic enquiry did not reveal any complaints and the systemic examination was normal.

#### Clinical findings

Local examination showed a swollen and tender left shoulder down to the mid-upper arm. Range of motion was largely reduced but the neurovascular status of the limb was preserved. Systemic examination of the patient was normal, with normal breathe sounds and a soft, non-tender abdomen.

#### Diagnostic assessment

**Fig. 3:**
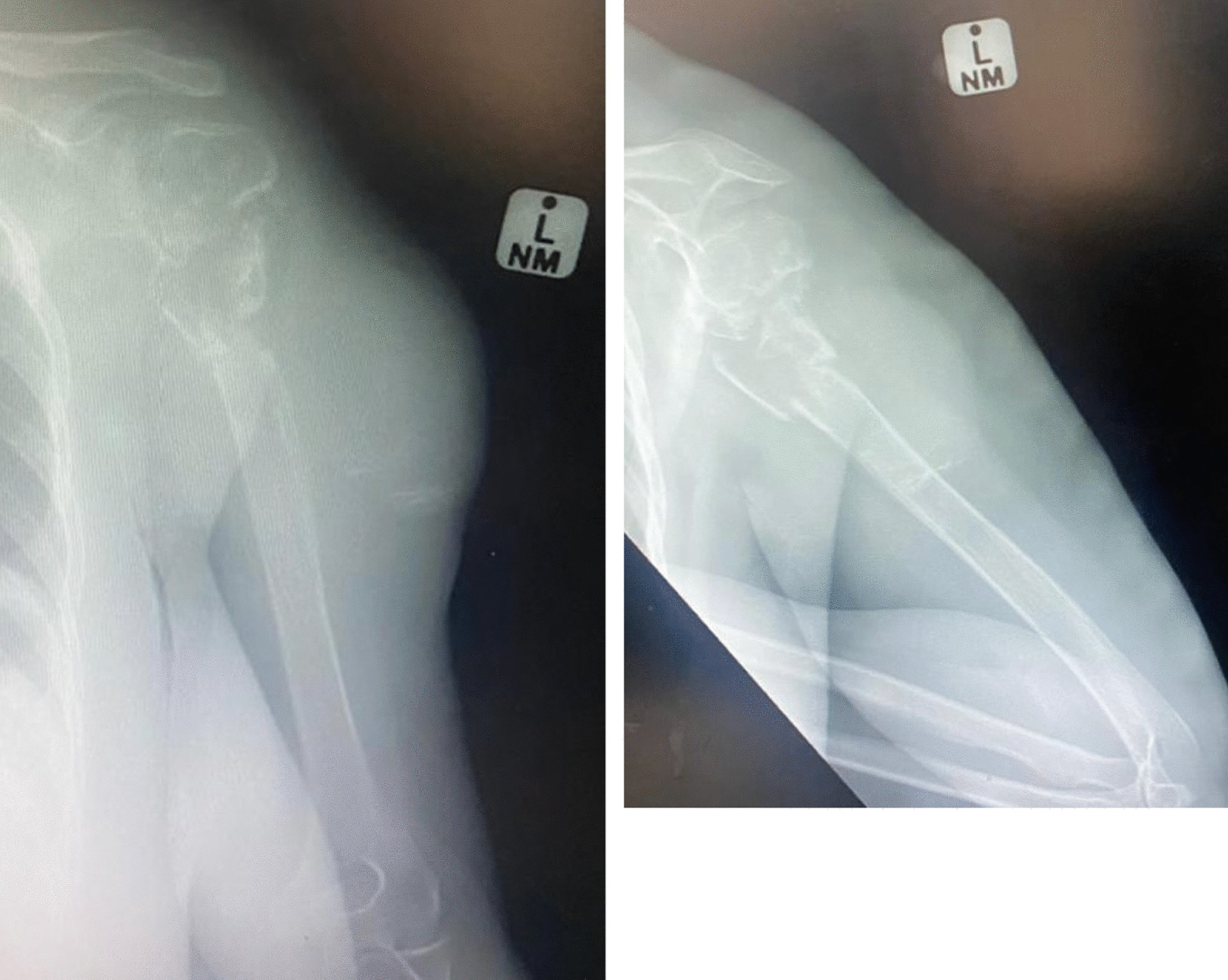
Pre-operative AP and lateral views of the left humerus

Pre-operative radiographs of the left shoulder are as depicted in Fig. 3 showing multiple lytic lesions of the proximal humerus and a pathological fracture. Subsequently an MRI was done and biopsy performed. This confirmed presence of laminated eosinophilic structure accompanied by foreign body giant cell response and morphological features in keeping with hydatid cysts. No serological work-up for hydatid cyst (enzyme-linked immunosorbent assay, complement fixation test) was done at any stage.

#### Therapeutic intervention

Because of her young age, the patient was offered joint-preserving surgery in the form of a cemented proximal humerus plate. This was combined with oral anti-parasitic treatment. She received both adjuvant and neoadjuvant therapy with albendazole 10 mg/kg/day in divided doses for four weeks before the surgery was performed and this was continued for a further 8 weeks after surgery.

#### Outcome

**Fig. 4:**
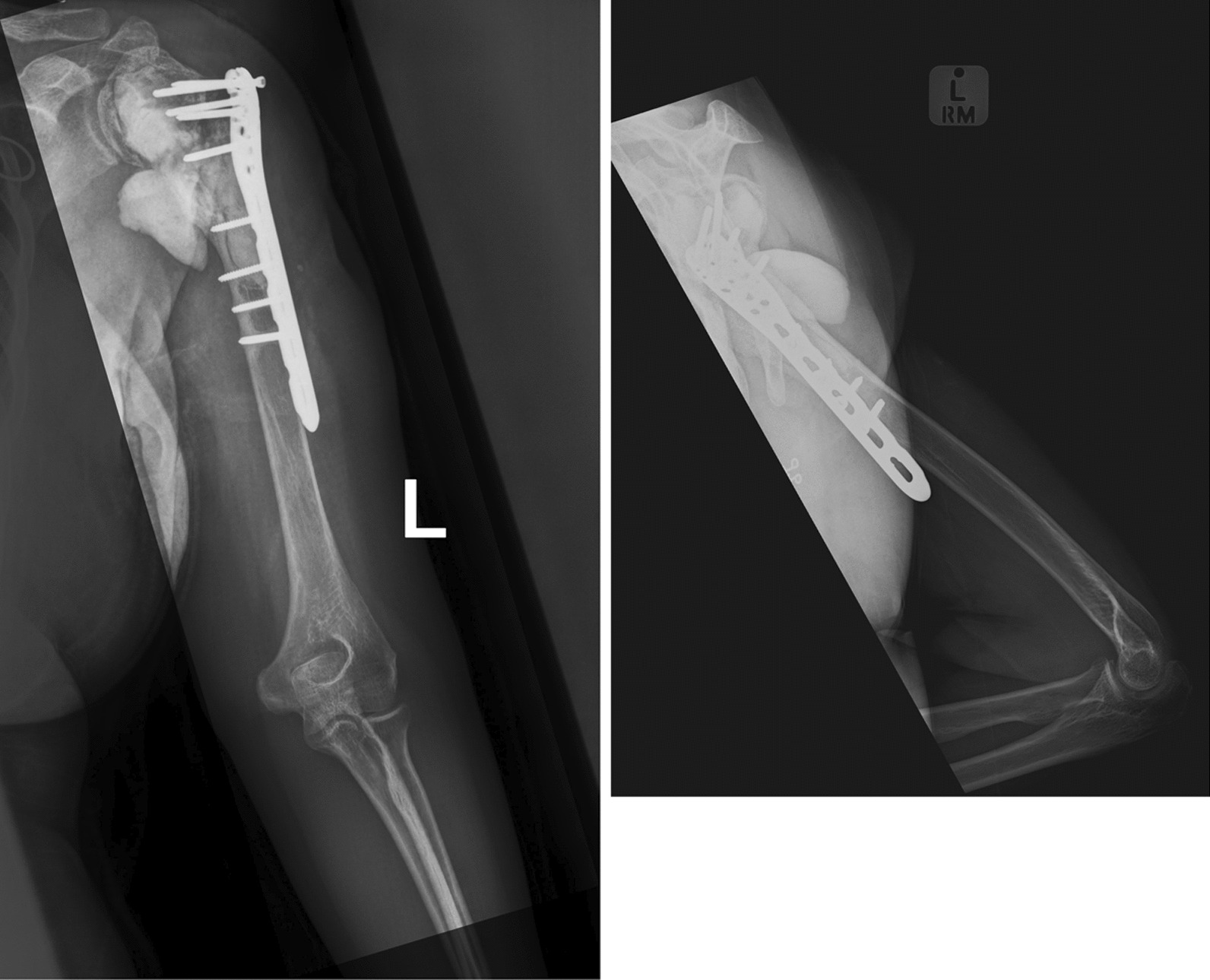
Two year-post-operative AP and lateral views of the left humerus showing implant failure

**Fig. 5:**
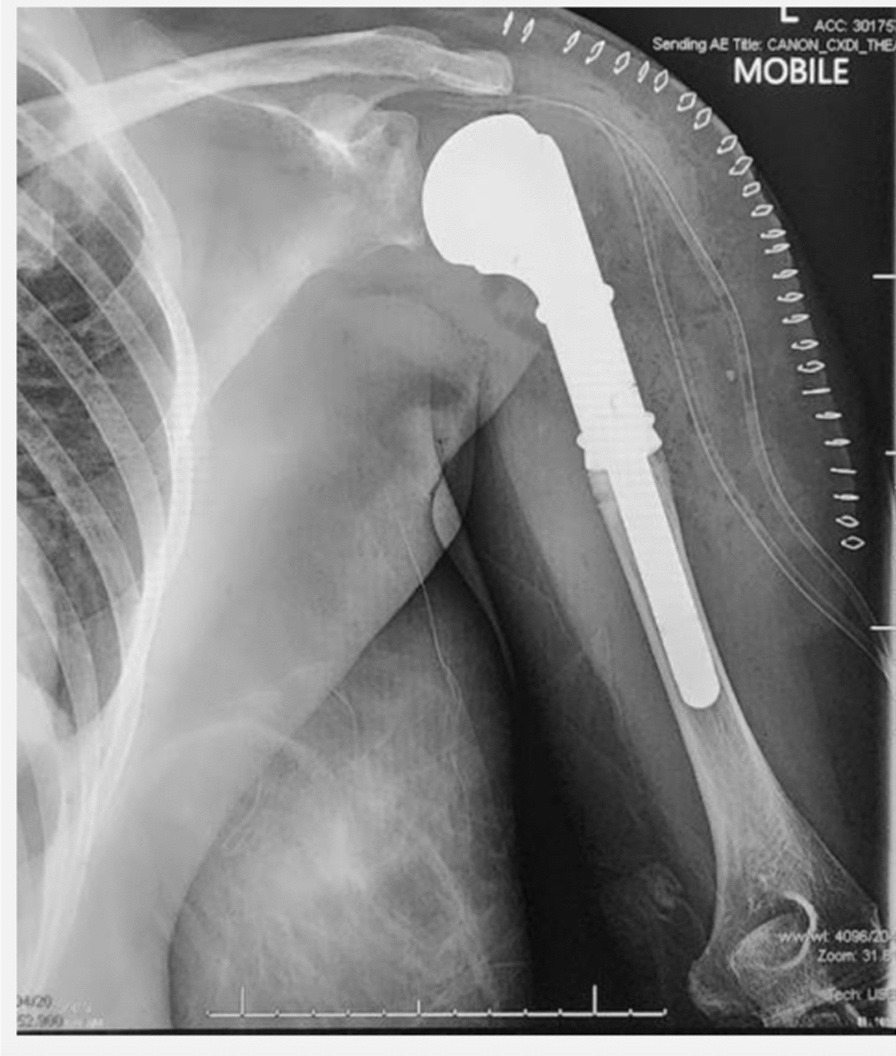
Post-operative AP and Lateral radiographs of the left shoulder after revision arthroplasty

At her 2-year follow-up radiographs showed significant hardware failure with screw loosening and the plate being displaced off bone (see Fig. 4). She is currently 39 years and has since undergone revision surgery to a shoulder arthroplasty (see Fig. 5).

## Discussion

Neelapala *et al.*, reported on a similar case of recurrent pelvic hydatid disease in a 35-year-old female that ended up with a THR [2]. Their case and ours demonstrate the elusiveness of the diagnosis of the disease in the musculoskeletal system. Patients are often subjected to multiple investigative efforts, prolonged treatment periods and multiple surgeries. The surgeries are very complex because of the degree of bony involvement by the disease and the duration of the disease at the time of surgery.

The choice of which surgery to perform is not a simple one and no gold standard exists to guide this decision. We opted for a cemented locked proximal humerus plate in case 2 because she was a young female at the time of presentation and we thought this would buy her valuable time before requiring a joint arthroplasty. Adam reported another case report of pelvic hydatid disease in a 49-year-old female and this patient went on to be lost to follow-up before any orthopaedic surgical intervention could be performed [7]. Worth noting is that many of the patients in these case reports, as in ours, are young females under the age of 40 years at first presentation [2, 7, 8]. The disease does predominantly affect females with a mean age of 42 years [1]. The reporting on outcomes in the literature is diversified. Bhatnagar *et al.*, reported a good outcome in a 35 year old female that was disease-free after a hip excision arthroplasty while Notarnicola *et al.*, reported a poor outcome for a patient that had multiple prosthetic hip dislocations [8, 9]. Siwach *et al.*, reported a death in a 51 year old female from sepsis from hydatid disease of the proximal femur and pelvis [10]. Our experience is that this is a very difficult disease to manage, recurrence is high and the prognosis after surgery is guarded at best. Patients will often need more than one surgery and the impact on their personal and professional life is grave. These are normally young and productive individuals, which adds a significant economic burden on their lives. Musculoskeletal hydatid disease is rare and of the known cases, 21% are of the pelvis [11]. Even rarer are that of the humerus. Patino and Vertiz performed a total humerus resection for a patient with total humerus involvement and reported good results following this however, this disease presents treatment challenges as it is difficult to develop standard protocols for surgical treatment on such low numbers and every case has to be individualised [12].

## Conclusion

Musculoskeletal hydatid disease is a rare condition and a difficult one to manage. The disease commonly affects young and productive females and treatment should aim to afford them a return to productivity. The diagnosis can be challenging to make and delays in treatment occur. There are no standardised orthopaedic surgical guidelines for its treatment and every case must be individualised, and a more radical surgical approach may be required from the outset.

## Data Availability

All data generated and analysed during this study are included in this published article..

## Abbreviations

HIV: Human immunodeficiency virus
MRI: Magnetic resonance imaging
TB: Tuberculosis
THR: Total hip replacement
